# Public engagement in health technology assessment in Brazil: the case of the Trastuzumab public consultation

**DOI:** 10.1186/s12913-019-4555-6

**Published:** 2019-10-28

**Authors:** Viviane Karoline da Silva Carvalho, Maria Sharmila Alina de Sousa, Jorge Otávio Maia Barreto, Everton Nunes da Silva

**Affiliations:** 1 University of Brasília, Brasília, Brazil; 2 Oswaldo Cruz Foundation, Brasília, Brazil

**Keywords:** Social participation, Public opinion, Public consultation, Health technology assessment (HTA), Public engagement, Analytical methods

## Abstract

**Background:**

Public engagement in health technology assessment (HTA) is increasing worldwide. There are several forms of public engagement and it is not always possible to determine which stakeholders participate in the HTA process and how they contribute. Our objective was to investigate which types of social representatives contributed to the public consultation on the incorporation of Trastuzumab for early-stage breast cancer treatment within the public health system in Brazil, held in 2012 by the National Committee for Health Technology Incorporation (CONITEC).

**Methods:**

A mixed methods approach was used to analyze social representativeness and the composition of the corpus from the public consultation, which consisted of 127 contributions. Three types of analysis were performed using IRaMuTeQ software: classic lexical analysis, descending hierarchical classification and specificities analysis. The contributions were clustered according to the main categories of discourse observed, into four social representation categories: 1) patient representation/advocacy; 2) pharmaceutical industry/advocacy; 3) healthcare professionals; and 4) individual contributions.

**Results:**

Category 1 contained words related to increased survival due to use of the drug and a low score for words pertaining to studies on Trastuzumab. The word “safety” obtained a positive score only in category 2, which was also the only category that exhibited a negative score for the word “risk”. Category 3 displayed the lowest scores for “diagnosis” and “safety”. The word “efficacy” had a negative score only in category 4.

**Conclusions:**

Each category exhibited different results for words related to health systems and to key concepts linked to HTA. Our analysis enabled the identification of the most prominent contributions for each category. Despite the promising results obtained, further research is needed to validate this software for use in analyzing public contributions.

## Background

The number of initiatives promoting social engagement in health technology assessment (HTA) has grown in recent years, [1–3] largely due to efforts by patient organizations and the HTA community [4, 5]. Fostering public engagement (on the part of users or interested citizens) can contribute to encouraging social responsibility, participatory democracy and transparency [4, 6]. Patients can provide unique additional perspectives for decision making, with experience-based insight on the benefits and disadvantages of using certain health technologies [4, 7].

The outcomes of determining and assessing patient engagement in HTA can be difficult to identify, quantify and qualify because of the social participation strategies implemented by HTA agencies [4]. However, assessment methods developed by these agencies to incorporate these social contributions remain unclear, making it difficult to determine how and when these contributions effectively explain the decision-making process in HTA [8].

Public engagement strategies employed by HTA agencies comprise a wide variety of actions, ranging from prioritizing research issues and gathering or analyzing evidence to public consultation on strategies for disseminating recommendations [3, 5]. Available publications on examples of experiences of public engagement in HTA indicate that the perspective of health system users can add important dimensions to the decision-making process within HTA [8]. In Brazil, society can be present in several stages of the process of evaluating and incorporating technologies into the National Health System (SUS), acting as consumers of technology incorporation, participating in public consultations or hearings, or taking part in CONITEC (National Committee for Health Technology Incorporation) board meetings as patient representatives [9, 10]. In 2015, CONITEC also adopted a strategy aiming at improving the understanding of general public on HTA evidence by means of plain-language summaries called report to society (*relatório para sociedade* in Portuguese).

Public consultations are the main public engagement mechanism used by CONITEC. Contributions are obtained via online public consultations on an issue and subsequently compiled, analyzed and presented to the CONITEC board for incorporation into the committee’s final recommendation which, like the public consultation, is available in its entirety on the CONITEC website, ensuring publicity and transparency, broadening the debate on certain issues and supporting decisions related to formulating and defining public health policies [10].

### Case study: Public consultation on incorporating Trastuzumab into the SUS

CONITEC is responsible for HTA at the Ministry of Health level in Brazil, whose public health system guarantees universal and equal access to comprehensive care through the National Health System (SUS) [11]. Created in 2012, CONITEC follows the same technology incorporation process as other countries with public health systems, such as France, the United Kingdom and Canada, fostering the use of clinical protocols, therapeutic guidelines and scientific evidence, for example, as well as social participation in the assessment process [9, 12]. In Brazil, any person or institution can request the incorporation of technology provided that the applicant submits studies to CONITEC corroborating its safety and efficacy, in addition to cost-effectiveness analysis and budget impact analyses [10]. After analysis and issuing a report, CONITEC’s recommendations are submitted to a public consultation, which will form part of the committee’s final report. CONITEC has 180 days to issue its final recommendation for ratification or not by the Secretariat of Science, Technology and Strategic Inputs, which can request a public hearing before making a decision [10].

In this respect, we opted for analyzing the public consultation on incorporating the drug Trastuzumab into early-stage breast cancer treatment in Brazil, held by CONITEC in 2012. This public consultation was chosen because Trastuzumab was one of the first drugs incorporated by CONITEC and to be the subject of a public consultation to assess technology incorporation in Brazil. Trastuzumab was included in the SUS in 2012, recommended for the treatment of HER2-positive early-stage breast cancer after surgery, chemotherapy (neoadjuvant or adjuvant) and radiotherapy (when applicable), requiring confirmation of HER-2 status before treatment [13]. There is solid scientific evidence favoring the use of Trastuzumab to treat women with HER2-positive breast cancer [14].

During the call for contributions to the public consultation on including Trastuzumab for early breast cancer treatment, CONITEC published a technical report about incorporating the drug. At that time, there were no documents available in lay terms to inform the general public about CONITEC’s recommendation. This changed in 2015, with the compiling of short, straightforward technical reports targeting the general population, stimulating public participation in the HTA process [10, 15].

Our aim is to propose a step-by-step tool to analyse the public consultation carried out by CONITEC. Our proposal applies a systematic and transparent process to review all the contributions raised by the public about a decision of inclusion/exclusion of a technology in the health system, by which a recommendation synthesis could be draw in an objective and timely manner. These attributes are essential to ensure high quality information from the public perspective in the decision-making process, particularly when the public consultation having hundreds of contributions, resulting in a difficult task to analyse manually. The recommendation synthesis would rely on four questions: i) is there convergence/divergence of opinion among different discourse categories about the technology under consultation? ii) is there public support for including/excluding the technology under consultation? iii) which are the main pros/cons arguments raised by the public about the inclusion/exclusion of the technology under consultation? iv) what are the main HTA evidence used by the public? This study is relevant to assess whether a step-by-step tool, including IRaMuTeQ software, could contribute to better delivery synthesis from the public perspective.

## Methods

A mixed methods approach [16] was used to qualify the typology of social representations and the composition of the corpus of all the contributions to the public consultation studied. We carried out a grounded theory-inspired case study to describe the types of discourses that could be identified by deploying IRaMuTeQ as a tool to transparently and systematically analyse complex corpus linguistics (textual data) from questionnaires and forms used in public consultations, as this developed by the HTA agency of Ministry of Health of Brazil. Triangulating content analysis [17] with the lexical analysis performed using IRaMuTeQ software (*Interface de R pour les Analyses Multidimensionnelles de Textes et de Questionnaires*) [18] allowed us to better explore the layers of meaning in the contributions, in order to provide a multifaceted answer to our research question [19]. IRaMuTeQ uses Python language as well as functionalities provided by the statistical software R [18].

The lexical analysis uses statistical methods to describe a vocabulary, showing general characteristics of the corpus such as the number of words occurrences, word forms and text segments considered in the analysis (retention) [17]. As a suggestion, these parameters can be used as an indicator for reproducibility, when other researchers replicate the analysis. Content analysis is a set of communication analysis techniques that, besides being able to make use of multiple collection instruments, can also be applied in several fields that include the emission of meanings from one sender to another, establishing a communication [17].

We selected IRaMuTeQ as a lexical analysis tool because it is free software capable of performing several types of statistical analyses on categories of actors/emitters and corpus/words [18]. Software such as IRaMuTeQ enables larger and more complex databases to be analyzed, giving researchers greater detail to explore, describe and compare data [20]. Thus, we exploited its potential to systematize and ensure transparency when analyzing the public consultations held by CONITEC, since there is no consolidated methodology for this type of assessment on the committee’s website. As such, our study is the first to use this innovative approach to systematize a methodology that qualifies contributions to a public consultation into classes of actors and words, as a form of social participation in HTA processes.

### Study design

The analysis was conducted in four stages. In the first stage, we considered the entire text as analytical material, exactly as it appeared in the CONITEC report. In the second, professional categories were grouped together in order to combine categories that were similar, but written differently (e.g. doctor and medical oncologist). This procedure was done according to software recommendations. Stage three was performed by identifying the social representations that emerged from the public consultation, based on the theory of social representations (TSR) developed by Serge Moscovici [21]. Based on that, we conducted an interpretive analysis of the corpus (stage three) and created a new variable (‘discourse category’), clustering the contributions according to the main categories of discourse observed: 1 - patient representation/advocacy; 2 - pharmaceutical industry/advocacy; 3 - healthcare professionals; and 4 - individual contributions. The contributions were assigned to categories by independent researchers (VKSC and MSAS) to reduce the risk of allocation bias. Based on the categories of discourse performed, we seek to know if these groups have similar arguments related to the incorporation of Trastuzumab.

In the final stage, we used IRaMuTeQ software to conduct three types of analysis: classic lexical analysis, descending hierarchical classification (DHC) and specificities analysis (specificities and factorial correspondence analysis – FCA). Lexical analysis involves organizing and counting the words in the vocabulary used, analyzing them and applying statistical methods to describe the dimension of the answers [22]. Descending hierarchical classification categorizes data according to the variables selected (‘discourse category’ in our case) [23]. It is based on the idea that words used in a similar context are associated and form part of specific representation systems, that is, it clusters text segments (TS) with similar vocabulary and separates those with different vocabulary [24]. The word classes generated by DHC were named based on the composition of the words and discourse in each class. Finally, using the software, we extracted excerpts from the corpus that were representative of each word class. Additionally, based on specificities analysis (which associates text with words) and FCA (which generates a graphical representation on a Cartesian plane), we analyzed the score of the words related to health systems and the assessment of health technology, using them for additional analyses of the discourse categories. Factorial correspondence analysis (FCA) involves crossing word classes and frequency using the chi-squared (χ^2^) correlation and frequency values of each word in the corpus to make it easier to visualize proximity (or distance) between the classes [24]. The chi-squared test (χ^2^) was applied in all the available analyses performed by the software, since it expresses the strength of the association between classes and words and its scores indicate the probability of correlation between the corpus variables and words [25]. The higher the score, the stronger the association between the word form/word and the word class (or discourse category).

Analyses were performed in the original language of the contributions (Brazilian Portuguese) and the results were translated into English.

### Data set

The data analyzed are from the public consultation on incorporating Trastuzumab into early-stage breast cancer treatment, held in 2012 on the CONITEC website. There were 127 contributions from several different states. Up to 3 contributions were permitted per person and the fields participants were required to complete (and were adopted as variables in the present study) were: state; municipality; occupation; type of institution; how you found out about the public consultation; and description of the contribution. Most of the contributions came from the states of São Paulo, Rio Grande do Sul e Minas Gerais.

### Ethical aspects

A secondary analysis of existing public data was carried out, where public consultations was published on the CONITEC’s website, but without publishing personal data of the participants. The public consultation was conducted by CONITEC and did not require approval by the Brazilian Research Ethics Committee, because it did not characterize a type of research with human beings.

Given that secondary data were used, where the public consultation can be classified as a public opinion survey with unidentified participants and in the public domain, this study did not have to be submitted to the Research Ethics Committee, in accordance with Resolution 510/16 of the Brazilian National Health Council [26].

## Results

### Characteristics of the Corpus

The corpus consisted of 114 texts, separated into 685 text segments (TS), 542 of which were used, corresponding to 79.12% of the total. The retention of 79.12% of the text segments emerged from the descending hierarchical classification, using the chi-squared test to classify different patterns of vocabulary in terms of their co-occurrences, pairs of words and sentence that are statistically frequently associated to classes of discourse [27]. Authors have suggested a minimal retention between 70 and 75% of the text segments for descending hierarchical classification be efficient [28], which we achieved in our study. There were 22,699 word occurrences, with 1914 different word forms and 646 words (2.85% of the total occurrences) that occurred only once (Table 1).

**Table 1:** Characterization of the corpus

Corpus	No. of Texts	No. of TS	No. of Occurrences	No. of Word Forms	No. of Lemmata	No. of Active Forms	No. of Supplementary Forms	No. of Hapaxes	TS Classification
Public Consultation on incorporating Trastuzumab for early breast cancer	114	685	22,652	1914	1469	1253	206	646	542 TS (79.12%)

LEGEND: No. of Texts: number of texts in the public contributions

No. of TS: number of text segment fragments identified by the software based on the number of texts

No. of Occurrences: total number of word occurrences

No. of Word Forms: number of word forms present in the text

No. of Lemmata: number of types related to headwords

No. of Active Forms: the main words in the corpus

No. of Supplementary Forms: words considered supplementary in the corpus

No. of Hapaxes: words that appear only once in the entire corpus

TS Classification: number of text segments used by the software

Source: compiled by the authors based on data obtained in IRaMuTeQ software

**Fig. 1:**
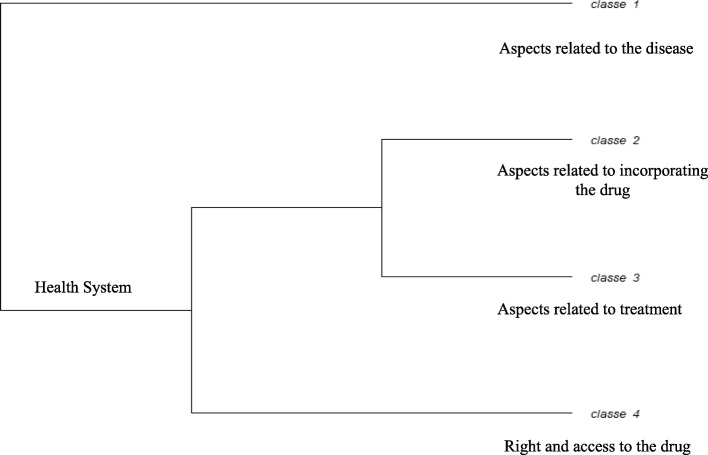
Main classes and subclasses resulting from DHC of the corpus. Source: adapted from the dendrogram obtained on IRaMuTeQ software

The content analyzed was categorized into four word classes (Fig. 1): class 1 (Aspects related to the disease - clinical study evidence), with 186 TS (34.32%), class 2 (Aspects related to incorporating the drug) with182 TS (33.58%), class 3 (Aspects related to treatment - MEDICATION), with 80 TS (14.76%), and class 4 (Right and access to the drug), with 94 TS (17.34%). These were divided into two branches, with three sub-branches: subcorpus *A* (class 1), subcorpus *B* (classes 2 and 3) and subcorpus *C* (class 4).

A table was compiled listing the main words and sentences in each word class (Table 2). All the excerpts were extracted based on all the words in the class. The absolute score is calculated based on the sum of the χ^2^ values of all the words in a class. Up to 50 TS were displayed.

**Table 2:** Main words and sentences per word class – Classes 1 to 4

CLASS 1 (34.32%) - Aspects related to the disease (clinical study evidence)			CLASS 2 (33.58%) Aspects related to incorporating the drug			CLASS 3 (14.76%) – Aspects related to treatment (MEDICATION)			CLASS 4: 94 TS (17.34%) – Right and access to the drug		
Cat. 4 – individual cont. (χ^2^ 128.39)			Cat. 2 – Pharmaceutical Ind. (χ^2^ 132.26) and Cat. 3 Health Prof. (χ^2^ 3.7)			Cat. 3 – Health Prof. (χ^2^ 48.35) and Cat. 1- Patient Rep./adv (χ^2^ 14.55)			Cat. 4 – individual cont. (χ^2^ 32.95)		
Main Words	χ^2^	Illustrative Excerpt	Main Words	χ^2^	Illustrative Excerpt	Main Words	χ^2^	Illustrative Excerpt	Main Words	χ^2^	Illustrative Excerpt
Breast Cancer	272.95	*“however more than 60 of these women will die from recurrence of the disease in other organs. Three types of systemic adjuvant postoperative therapy have proved to be effective at significantly lowering the chance of breast cancer recurring after curative surgery”*	Herceptin	52.29	*“position of the Oncoguia institute regarding the CONITEC document that addresses the approval of Trastuzumab for early-stage breast cancer. Herceptin Trastuzumab is indicated for the treatment of patients with early-stage her2-positive breast cancer either before surgery as neoadjuvant treatment or after surgery as adjuvant treatment”*	Disease-Free Survival	157.72	*“and subsequently as an isolated drug or concomitantly to hormone therapy until completing a total of one year of treatment. The change in the text is justified by the fact that, based on studies published to date, Trastuzumab concomitantly to chemotherapy exhibits better results than when the drug is administered only after chemotherapy”*	National Health System	383.38	*“additionally, according to the studies mentioned, it is recommended that Trastuzumab be administered for a period of one year and as such, Femama believes that national health system patients should receive the complete treatment in the same manner as patients with health insurance plans”*
Disease	178.77		Patient	51.29		Overall Survival	126.16		Incorporation	259.1	
Death	124.75		Clinical	38.95		Change	113.71		Health Insurance	124.91	
Malignant	112.44		CONITEC	36.83		Arm	113.71		Indisputable	118.33	
Organ	107.75		Recommendation	35.12		Docetaxel	95.21		Coverage Type	112.24	
Significantly	103.74		Data	34.72		Paclitaxel	89.09		Complete	106.6	
Metastasis	102.21		Indicated	30.51		Text	76.92		Benefit	88.47	
Occurrence	102.21		Roche	28.43		Justify	76.92		Medication	87.11	
Rationale	101.41		Consider	27.58		Isolated	63.66		Brazil	84.63	
Molecular	101.41		Safety	26.35		No	61		Need	73.02	
Recurrence	93.82		Cross	26.35		Chemotherapy	60.41		Duty	53.99	
Risk	90.22		Her2-Positive	25.54		Difference	52.85		Femama	44.34	
Development	85.07		Presentation	24.27		Demonstrate	50.37		Take	28.92	
Surgery	82.03		Diagnosis	24.27		Regime	46.89		Add	28.92	
Women	68.19		Adverse	24.27		Complete	46.89		Reversible	28.92	

Source: compiled by the authors based on data obtained in IRaMuTeQ software

**Fig. 2:**
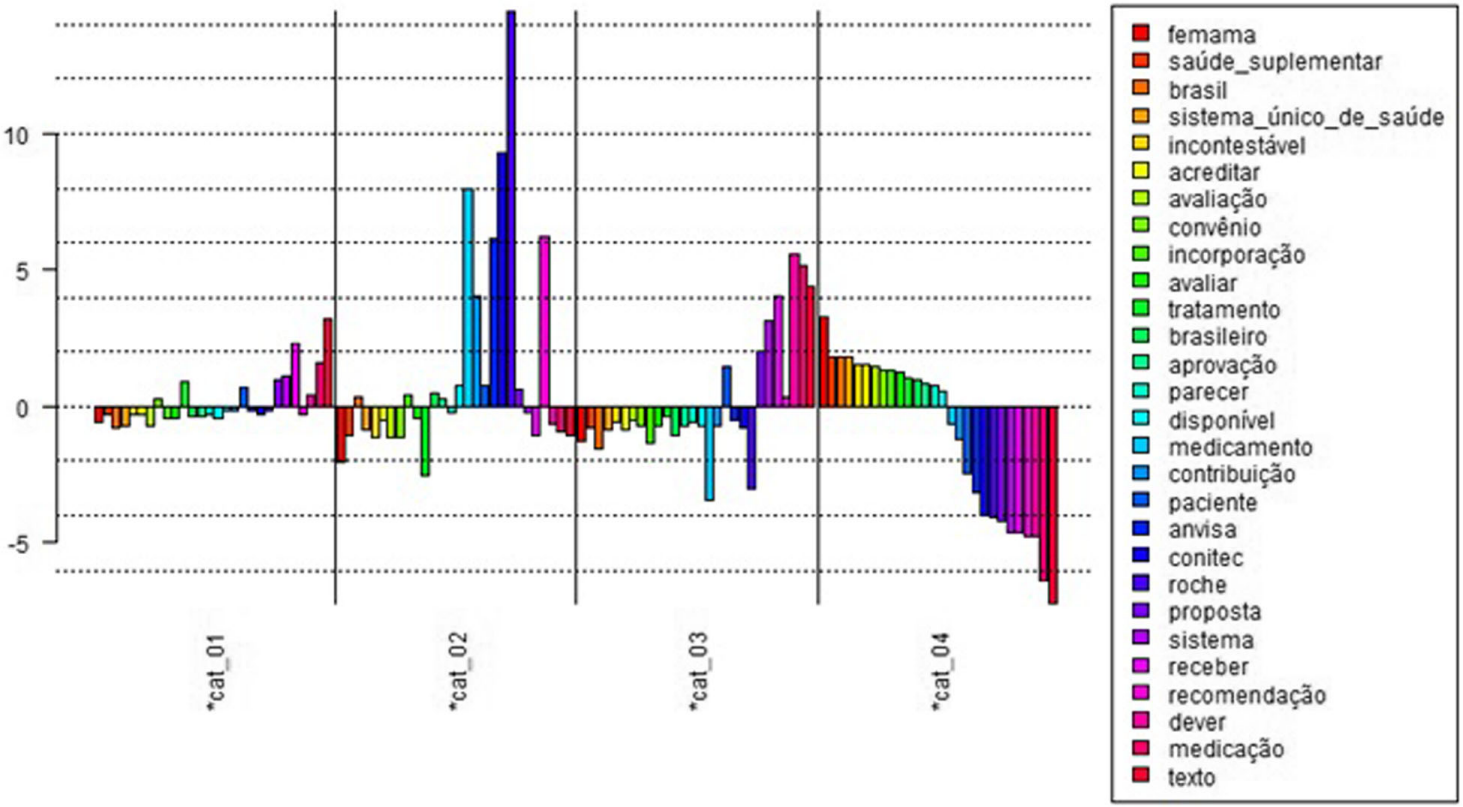
Graph 1 - Distribution of words related to ‘health system’ by ‘discourse category’. LEGEND: *Cat_1: patient representation/advocacy. *Cat_2: pharmaceutical industry/advocacy. *Cat_3: health professionals. *Cat_4: individual contributions. Source: compiled by the authors based on an analysis performed in IRaMuTeQ software . Notes: From the top to the bottom – femama, health insurance, Brazil, national health system, indisputable, believe, evaluation, reimbursement, incorporation, evaluate, treatment, Brazilian, approval, report, available, medication, contribution, patient, Anvisa, Conitec, Roche, proposal, system, receive, recommendation, duty, medication, text

Specificities analysis and FCA of word distribution in ‘discourse categories’ focused on words related to the health system and key concepts, as well as those associated with health technology assessment. The scores (obtained by χ^2^) for ‘health system’-related words are shown in Graph 1 below (Fig. 2).

### ‘Health system’ corpus

The word ‘text’ obtained the highest score in category 1 (patient representation/advocacy) – that is, the χ^2^ calculation by IRaMuTeQ revealed a strong (statistical) association between this discourse category and the word ‘text’, which refers to the changes needed to the text in CONITEC’s initial technical report about incorporating Trastuzumab for early breast cancer treatment. The words with the lowest score in this category were ‘Brazil’, ‘National Health System’ and ‘assessment’.

Category 2 (pharmaceutical industry/advocacy) exhibited the greatest differences in distribution when compared to the other categories: it was the only category in which the highest scores were concentrated in the middle of the graph, obtained for the words ‘recommendation’ (related to CONITEC’s recommendation report), ‘Roche’ (the company that manufactures Trastuzumab), ‘CONITEC’ and ‘Anvisa’ (the National Health Surveillance Agency). The lowest score was for the word ‘evaluate’ (referring to aspects involved in evaluating the drug), followed by ‘Femama’, the Brazilian Federation of Philanthropic Breast Health Supporting Organizations.

The highest scoring word in category 3 (health professionals) was ‘duty’, which referred to aspects such as SUS patients’ right to have access to medication, monitoring of the drug by the Ministry of Health, how the necessary tests and treatments using the drug should be carried out, the fact that cardiac toxicity should not be a limiting factor in administering the drug, but should be added to the direct costs and offset by managers when making decisions regarding incorporating the medication. Therefore, a ‘duty’ of the State.

In category 4 (individual contributions), which contains the contributions that the software was unable to classify into the previous categories, the word ‘Femama’ obtained the highest score and was related to the technical report published by Femama on Trastuzumab for HER2-positive breast cancer. The lowest score recorded was for ‘text’. The graph indicates a contract between distribution in categories 1 and 2, where almost all the words associated with the health system that were selected and exhibited a positive score in category 1 obtained a negative score in category 2 (and vice versa).

### ‘HTA’ corpus

**Fig. 3:**
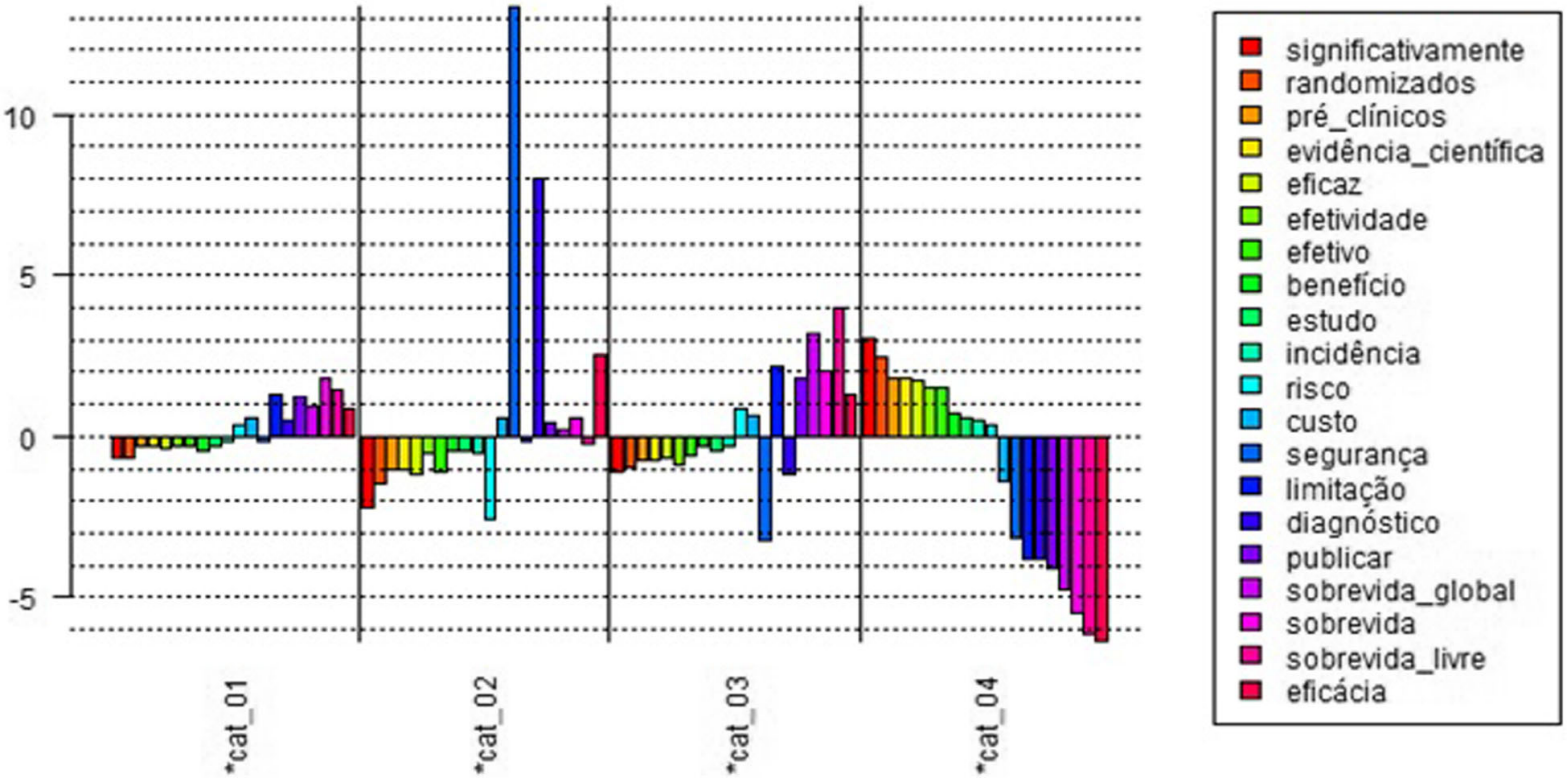
Graph 2 - distribution by ‘discourse category’ of words referring to key concepts related to ‘health technology assessment’. LEGEND: *Cat_1: patient representation/advocacy. *Cat_2: pharmaceutical industry/advocacy. *Cat_3: health professionals. *Cat_4: individual contributions. Source: compiled by the authors based on an analysis performed in IRaMuTeQ software. Notes: From the top to the bottom – significantly, randomized, preclinical, scientific evidence, efficacious, effectiveness, effective, benefit, study, incidence, risk, cost, safety, limitation, diagnosis, publish, overall survival, survival, disease-free survival, efficacy

We selected 20 words that focus on key concepts and words related to HTA. Initial findings indicate similar ‘discourse category’ distribution among words referring to the ‘health system’ and key concepts and those related to ‘HTA’, as are shown in Graph 2 below (Fig. 3).

In category 1 (patient representation/advocacy), the words ‘significantly’ and ‘randomized’ obtained the lowest scores. In the corpus, these words referred to the types of results that can be achieved with Trastuzumab, and research on Trastuzumab, respectively. The highest scoring words were ‘survival’ and ‘disease-free survival’. ‘Survival’ was present in contributions that indicated patients had experienced increased survival by using Trastuzumab. A significant portion of the contributions containing this word stated that because patient survival improved, the time until brain metastases also increased, indicating a good prognosis due to the use of Trastuzumab.

The lowest score in category 2 (pharmaceutical industry/advocacy) was for ‘risk’, followed by ‘significantly’. ‘Safety’ is associated with the safety of patients using the medication and exhibited the highest score. In fact, it obtained a positive score only in this category.

The second highest scoring word was ‘diagnosis’, which also obtained a positive score in category 1 and is related to the tests and methods used to diagnose HER2 status.

In research, the word ‘limitation’ typically refers to items that could not be achieved or restrictions during the study. This word obtained the highest score in the categories patient representation/advocacy (category 1) and health professionals (category 3).

In category 3, the highest scores were recorded for ‘disease-free survival’, ‘overall survival’ and ‘limitation’, with ‘safety’ and ‘diagnosis’ obtaining the lowest scores.

Finally, the highest scoring words in category 4 (individual contributions) were ‘significantly’ and ‘randomized’, in contrast to their scores in category 1, with the same graphic representation as the words related to ‘health system”. ‘Efficacy’ was the lowest scoring word in the category and displayed a positive score in all the other categories analyzed.

## Discussion

Public engagement strategies should be devised in conjunction with society, allowing the public to choose their preferred form of engagement in HTA and bridging the gap between society and healthcare decision-making processes [2]. When developing healthcare guidelines in the United Kingdom, for example, the National Institute for Health and Care Excellence (NICE) stipulates that at least two ‘consumers’ of the guideline in question be involved in the process and provides information on the role of all participants [29].

In the United States, the so-called ‘golden triad’ of public engagement for defending patient rights consists of health professionals (who provide clinical information to support political arguments), patient advocates (who contribute with their experiences of a specific health condition and how policies affect them specifically), and government-relations professionals, who identify possible barriers and facilitators to policy formation [30]. In this study, in addition to the engagement of health professionals and patient advocates, the public consultation analyzed also featured contributions from the pharmaceutical industry and other interested parties from different sections of the Brazilian population, such as women with breast cancer, caregivers and/or citizens without the disease.

According to the public engagement model for compiling strategies to develop health services and technology [31], category 1 (patient representation/advocacy) was expected to contain a greater frequency of words directly linked to patient health needs. However, the words that express these needs (‘benefit’, ‘indisputable’, ‘Femama’) did not obtain positive scores, that is, were not relevant to this category. For instance, ‘Femama’ refers to a support organization for breast cancer patients and caregivers, and ‘benefit’ and ‘indisputable’ to the benefits of the medication and the needs for its incorporation to be irrefutable. The aforementioned patient engagement model supports the idea that the intervention would be better suited to patient needs, since it incorporates the views of stakeholders [31]. As such, the needs of participants in category 1 (‘patient representation/advocacy’) can de deduced through the words ‘text’, ‘survival’ and ‘disease-free survival’ by considering the aspects each word is related to.

The engagement of patients, their caregivers and patient advocates enhanced the public consultation process on Trastuzumab, since they are not required to be experts in HTA and sharing their experiences with decision makers may be decisive in confirming and/or changing opinions on basic healthcare issues [30]. However, there are certain limitations and consequences of public engagement; for example, when patient advocates have a strong personal connection to the cause they are defending it can affect their ability to examine evidence and compromise the HTA analysis process as a whole [29]. The different interests and prejudices (declared or not) of individuals in any of the categories may also influence decisions toward a path they might not have taken if based solely on scientific evidence [25, 28]. Nevertheless, these limitations do not mean that public contributions should not be heard, but rather that they should be considered in conjunction with other important factors, such as devising systematic, transparent, democratic and evidence-based social participation strategies for decision-making processes [31, 32].

While social actors can provide significant insight on certain health issues, they may also represent a conflict of intellectual and financial interests. According to McCoy et al. (2017), of the 104 most influential patient organizations in the United States, 83% receive funding from the pharmaceutical industry and 36% have a member of their industry on their board of directors [33]. This creates a deadlock in that patient contributions are important and difficult to replace despite the potential conflict of interest, making it a challenge to properly manage this conflict alongside individual contributions [29, 34].

For instance, the pharmaceutical industry can both positively and negatively influence the HTA process, with concerns that its engagement may lead to bias when assessing the evidence and a need for more time and resources [35]. In this respect, it is vital that each perspective be evaluated according to the context and considered in conjunction with scientific evidence and other social contribution strategies to the HTA process [32].

The pharmaceutical industry is among the most globalized and profitable in the world, and although doctors are still the primary target of its advertising and marketing initiatives, the internet has heightened its search for influence among the general public [30]. A critical point for advocates of public engagement is who is responsible for initiating engagement and who therefore decides the composition of the group that will participate in the process [31]. When analyzing a public consultation, it is important to carefully consider all the stakeholders involved to ensure a transparent assessment.

The highest scoring word in category 3 (health professionals) was ‘duty’, followed by ‘disease-free survival’, ‘overall survival’ and ‘limitation’. ‘Diagnosis’ and ‘safety’ were not expected to obtain the lowest scores since they are related to factors that are relevant to health professionals, whereas the safety of patients using Trastuzumab was expected to exhibit a positive score among these professionals. The word ‘duty’ in this category demonstrated that monitoring the drug is the duty of the Ministry of Health and that patients have a right to access it.

‘Significantly’ and ‘Femama’ achieved the highest score in category 4. A number of contributions in this category were based on the same Femama report, which may be why the word obtained such a high score. As several contributions used the Femama’s report, it may indicate a strategy of advocacy. The fact that this category contains different social actors reinforces the idea that although the members of a community may engage in issues related to their own or their family’s health (as in category 1, for example), they can also strive to address issues other than their own. This requires a greater collective effort, but allows communities to build their own identity and become involved in issues put forward by public institutions [20].

It is worth noting we have not carried out an investigation of potential conflict of interest when responders receive any financial support from a third party, since this information is not publicly available for all responders.

Another point is that the number of contributions from the public consultations carried out by CONITEC varies a lot depending on the health technology under decision. Some drugs, for example, mobilize more public engagement than others. For example, the Clinical Guideline for Multiple sclerosis [36] obtained 433 contributions while the clinical guideline for Compression Stockings for chronic venous insufficiency obtained only 17 contributions [37].

### Barriers and facilitators to using IRaMuTeQ to analyze Public consultations

The internet has provided new ways of keeping the public informed and increasing their contributions through interactive engagement. However, not all communities have access to the internet, which limits access to online public engagement platforms. As a result, the internet could further exacerbate inequalities in public engagement and should be used in conjunction with other strategies [6]. Following the example of low-income countries, radio could be used as a good mass media strategy to keep the public informed [6].

We were unable to find studies in the literature that analyzed public consultations on technology incorporation using IRaMuTeQ software, making ours the first to use this tool to ensure systematization and transparency when assessing contributions to public consultations held by HTA agencies such as CONITEC. We believe that using the software allowed us to obtain initial findings on biases in the contributions and, because it is automated, contributed to a systematized and transparent analysis. This helped reduce potential biases in the assessment of public consultations by those involved in HTA management processes.

Furthermore, we feel the software provides support in qualifying the typology of social representations involved in HTA contributions in Brazil, helping to create better strategies for identifying conflicts of interest and increasing engagement in all social classes.

We believe the use of IRaMuTeQ enables faster analysis of the types of arguments that are present in each category of social representation. This agility would be important for CONITEC, which has 180 days to complete the whole process of recommending a health technology. In a public consultation it is important to know which actors are participating in the consultation, as well as which actors are absent from participation. This information is important to know which social actors need to be engaged and, from that information, to think about how public engagement can be improved.

However, using software requires training and time in order to master the tool. Although IRaMuTeQ is user friendly, inadequate training and/or lack of timely technical support can result in performance errors that hamper its use. Another important aspect to consider is the time needed to prepare the corpus. This is a painstaking process because the corpus contains terms written in a variety of ways that need to be standardized (e.g. National Health System and its acronym SUS, which mean the same thing but are interpreted as different words by the software).

Additionally, the HTA agency should compile the collection form for public consultation in a way that facilitates systematization and subsequent analysis. We recommend using multiple-choice questions to characterize the participants and open-ended questions to obtain information about their experiences, perspectives, opinions and interests in order to identify the relevant social representations in each case. It would be important to include a declaration of potential conflict of interest from the responders. CONITEC should also discourage the cases where the responders copy/paste the same comment of others, since this information do not aggregate new point of view from the public, just increase the frequency of the same information.

Given that this is the first study to use IRaMuTeQ software for these analyses, further research is needed to ensure it is properly validated. The next step is to apply this approach to other study cases of public consultations from CONITEC, and eventually in other applicable cases from other HTA agencies abroad. Moreover, it is important to develop a method to validate our proposal of methodology, probably by triangulating other qualitative method to ensure the results are valid and pertinent to the decision-making process in HTA issues.

## Conclusion

This study applied a new approach to analyze the public consultation carried out by CONITEC in order to summarize the public contributions in a systematic, transparent, objective and timely manner. This approach could be useful to improve the technical capacity to systematize the contributions in another’s public consultations.

Based on that, we found four main results. First, we found convergence among the four categories of discourse in favor of the inclusion of Trastuzumab for HER2-positive early-stage breast cancer in the Brazilian public health system. Second, we did not identify arguments against the incorporation of Trastuzumab for HER2-positive early-stage breast cancer. Third, the main arguments in favor of Trastuzumab were: i) better outcomes when Trastuzumab is administrated in combination with other forms of chemotherapy; ii) the dosage unit should be the same as in other countries; iii) the risk of cardiac toxicity should not be a limiting factor for the use of trastuzumab in patients with normal cardiac function; iv) HER2-positive breast cancer is more aggressive and it has higher death risk, which justifies the inclusion of trastuzumab. Four, the most frequent HTA terms/concepts used by the contributors were safety (category of pharmaceutical industry), benefit, effectiveness and scientific evidence (category of individual contributions), efficacy (categories of patient representation/advocacy, pharmaceutical industry and health professionals) and risk (categories of patient representation/advocacy, health professionals and individual contributions).

## Data Availability

The datasets analysed during the current study are available in the CONITEC repository, [http://conitec.gov.br/images/Relatorios/2012/Trastuzumabe_caavancado_final.pdf.].

## Abbreviations

Anvisa: National Health Surveillance Agency
CONITEC: National Committee for Health Technology Incorporation
DHC: Descending hierarchical classification
FCA: Factorial correspondence analysis
Femama: Brazilian Federation of Philanthropic Breast Health Supporting Organizations
HTA: Health Technology Assessment
IRaMuTeQ: *Interface de R pour les Analyses Multidimensionnelles de Textes et de Questionnaires*)
NICE: National Institute for Health and Care Excellence
SUS: National Health System
TS: Text segments
TSR: Theory of Social Representations
χ^2^: Chi-squared
